# Case Report: Serial blood benzene and urinary phenol monitoring during extracorporeal treatment after severe acute oral ingestion of a benzene-containing solvent

**DOI:** 10.3389/ftox.2026.1875489

**Published:** 2026-08-20

**Authors:** Junting Liu, Quan Gan, Geng Zhang

**Affiliations:** 1 Department of Critical Care Medicine, Maternal and Child Health Hospital of Hubei Province, Wuhan, China; 2 Physics and Chemistry Laboratory, Hubei Provincial Hospital of Integrated Chinese and Western Medicine, Wuhan, China; 3 Hubei Provincial Clinical Research Center for Pneumoconiosis and Poisoning, Wuhan, China

**Keywords:** acute poisoning, benzene, biological monitoring, continuous veno-venous hemodiafiltration, hemoperfusion, oral ingestion, urinary phenol

## Abstract

**Background:**

Acute oral benzene poisoning is uncommon, and evidence to guide extracorporeal treatment and bedside toxicokinetic interpretation in this setting is limited. Most published experience concerns inhalational exposure or chronic hematotoxicity, leaving clinicians with little practical guidance for managing large intentional ingestions.

**Case Report:**

An 18-year-old man presented approximately 12 h after ingesting about 200 mL of a commercial benzene standard solution in ethyl acetate as intentional self-poisoning. On admission he had tachycardia, persistent gastrointestinal symptoms, and laboratory confirmation of exposure, with benzene detected in gastric fluid (2,966 μg/L), blood (457 μg/L), and urine (224 μg/L). Before gastric lavage, toxicological confirmation was obtained in all three matrices. After decontamination and supportive care, sequential hemoperfusion and continuous veno-venous hemodiafiltration (CVVHDF) were initiated. Serial toxicological monitoring showed rapid decline of the parent compound—blood benzene decreased to 228 μg/L at 20 h, 78 μg/L at 28 h, and became undetectable by 32 h—whereas urinary phenol rose later, peaking at 3231.5 mg/L at 32 h before declining over subsequent days. The clinical course included rhabdomyolysis, transient liver injury, erosive gastritis, and transient nonspecific white-matter abnormalities on brain magnetic resonance imaging (MRI). The patient recovered without persistent neurologic deficit and returned to usual activities.

**Conclusion:**

In severe acute oral ingestion of a benzene-containing solvent, paired monitoring of the parent compound and a major metabolite provided clinically useful information to contextualize extracorporeal treatment when formal decision thresholds were unavailable. This case cannot establish treatment efficacy, but it illustrates a pragmatic monitoring framework that may assist clinicians interpreting late toxicokinetic trends in rare but high-risk solvent ingestions.

## Introduction

Benzene is a volatile aromatic hydrocarbon widely used as an industrial solvent and chemical feedstock. Acute high-dose exposure may produce central nervous system depression, gastrointestinal injury, rhabdomyolysis, hepatic injury, hematologic abnormalities, and multiorgan dysfunction, and lethal acute poisonings have been reported (Agency for Toxic Substances and Disease Registry, 2024). Most published literature concerns inhalational occupational exposure or chronic hematotoxicity and leukemogenesis, whereas massive oral ingestion as intentional self-poisoning is uncommon and poorly characterized (Mohammed et al., 2020; Wang, C., et al., 2025).

Once absorbed, benzene is rapidly distributed into lipid-rich tissues and is extensively metabolized in the liver to more hydrophilic compounds, including phenol and its glucuronide/sulfate conjugates, as well as hydroquinone, catechol, and trans,trans-muconic acid (Agency for Toxic Substances and Disease Registry, 2024; Zhang et al., 2024). As a result, blood benzene alone may not fully reflect ongoing toxic burden in patients presenting several hours after a large ingestion, and biomarker selection depends on the exposure scenario and time course (Rahimpoor et al., 2023; Zhang et al., 2024).

Clinicians faced with a massive oral ingestion of a benzene-containing solvent encounter two practical problems. First, there are no benzene-specific recommendations from the EXTRIP workgroup, and broader principles for extracorporeal support in poisoning must be applied pragmatically (Ghannoum et al., 2018). Second, bedside biomarkers that can track evolving internal toxic burden after delayed presentation are not well defined in acute poisoning, in contrast to their established role in occupational biomonitoring at low exposure levels (Rahimpoor et al., 2023; Zhang et al., 2024). Dynamic toxicant monitoring has recently been highlighted as a clinically useful strategy in other acute solvent and pesticide poisonings managed with extracorporeal therapy (Liu et al., 2025; Wang, K., et al., 2025).

Here, we report a severe acute case of massive oral ingestion of a benzene-containing solvent in which serial blood benzene and urinary phenol measurements were used to contextualize hemoperfusion and CVVHDF during the acute phase. To our knowledge, this is the first detailed description of paired parent-compound and metabolite monitoring during hemoperfusion and CVVHDF after severe oral ingestion of a benzene-containing solvent.

## Case report

An 18-year-old man with no relevant medical or psychiatric history ingested approximately 200 mL of a commercial benzene standard solution as intentional self-poisoning. According to the product label, the solution contained benzene at 1 mol/L dissolved in ethyl acetate. The calculated ingested dose was approximately 15.6 g of benzene. He developed nausea and repeated vomiting within minutes of ingestion and reportedly lost consciousness transiently. Family members did not witness any seizure activity and brought him to the emergency department of Maternal and Child Health Hospital of Hubei Province approximately 12 h after ingestion.

On arrival, he complained of chest tightness, abdominal pain, nausea, retching, sore throat, and fecal incontinence. A strong solvent odor was noted in the vomitus. His heart rate was 140 beats/min, and electrocardiography showed sinus tachycardia with right axis deviation. After initial resuscitation, he was awake, cooperative, and oriented, with a Glasgow Coma Scale score of 14 and no focal neurologic deficit. He was able to maintain airway patency and protect his airway, and endotracheal intubation was not performed. Low-flow supplemental oxygen was administered. Bedside cardiac and pulmonary ultrasonography showed no major abnormalities. Initial laboratory testing showed leukocytosis (white blood cell count 20.85 × 10^9^/L) and mild coagulation abnormality (prothrombin time 14.8 s). Emesis and stool were positive for occult blood.

Before gastric lavage, toxicological confirmation was obtained in multiple matrices (gastric fluid benzene 2,966 μg/L, blood benzene 457 μg/L, urinary benzene 224 μg/L). Initial management then included skin decontamination, orogastric lavage with warm saline, and oral catharsis. Glucuronolactone, reduced glutathione, and vitamin C were administered by intravenous infusion as adjunctive hepatoprotective therapy. No N-acetylcysteine was administered. Apart from glutathione and vitamin C, no additional antioxidant therapy was given. Serial toxicological sampling together with urinary phenol monitoring was then initiated during the subsequent course.

Given the large reported ingestion, persistent measurable benzene in gastric fluid and blood 12 h after ingestion, and concern for evolving systemic toxicity, a sequential extracorporeal blood purification strategy was undertaken. Because the patient presented approximately 12 h after ingestion and initial decontamination procedures were completed first, HP was started immediately after completion of gastric lavage and catharsis, approximately 4 h after admission. HP was performed using an HA330-II disposable hemoperfusion cartridge (Jafron Biomedical Co., Ltd.) at a blood flow of 150 mL/min for 4 h per session under citrate regional anticoagulation, with sessions started at approximately 16, 28, 40, and 64 h after ingestion. CVVHDF was delivered using an Ultraflux AV600S polysulfone hemofilter (Fresenius Medical Care) and was prescribed at an effluent dose of approximately 30 mL/kg/h with equal diffusive and convective clearance (blood flow 130 mL/min; post-dilution replacement 1100 mL/h). The addition of CVVHDF at 28 h was intended primarily to support extracorporeal clearance of relatively water-soluble metabolites, particularly as urinary phenol increased during the later course and evolving systemic toxicity suggested ongoing metabolite burden (Ghannoum et al., 2018). This rationale was biologically plausible because phenol is substantially more water-soluble than benzene, whereas intermittent hemoperfusion was continued thereafter as part of the overall extracorporeal strategy because phenol does not behave as a purely hydrophilic molecule. Treatment decisions were not based on one concentration threshold alone; they integrated the reported ingestion amount, delayed presentation, persistent measurable benzene, evolving urinary phenol trends, ongoing gastrointestinal symptoms, dark urine, and laboratory evidence of systemic injury. De-escalation was based on the combined pattern of declining urinary phenol, clinical improvement, and improving laboratory markers. Other treatments included furosemide during the first 2 days, empirical antimicrobial therapy after chest imaging suggested a right lower lobe infection, and general intensive care support.

During the first 2 hospital days, the patient developed dark urine and evolving rhabdomyolysis. Creatine kinase rose from 54 U/L on admission to 3786 U/L on hospital day 1, and serum myoglobin rose to 1097.09 ng/mL. Liver injury became more apparent later, with alanine aminotransferase (ALT) and aspartate aminotransferase (AST) peaking at 330 U/L and 250 U/L, respectively, on hospital day 4. Chest and abdominal computed tomography (CT) on hospital day 3 showed a right lower lobe pulmonary infection and cholestasis. Brain MRI on hospital day 3 already showed scattered small white-matter lesions in the bilateral frontal and parietal lobes; similar nonspecific lesions were again seen on repeat MRI on hospital day 15 and later resolved on follow-up imaging. Gastroscopy on hospital day 15 showed erosive gastritis.

Clinical symptoms gradually improved during hospitalization. Dark urine resolved, muscle injury markers declined after their early peak, liver enzyme abnormalities improved, and mild late leukopenia resolved during follow-up. Follow-up brain MRI showed normalization of the earlier white-matter findings. After psychiatric evaluation and psychological support, the patient returned to a normal mental state. At 1-year follow-up, psychological status remained normal, and repeat clinical examinations and laboratory tests were unremarkable. The patient had returned to usual activities without new neurologic complaints.

**FIGURE 1:**
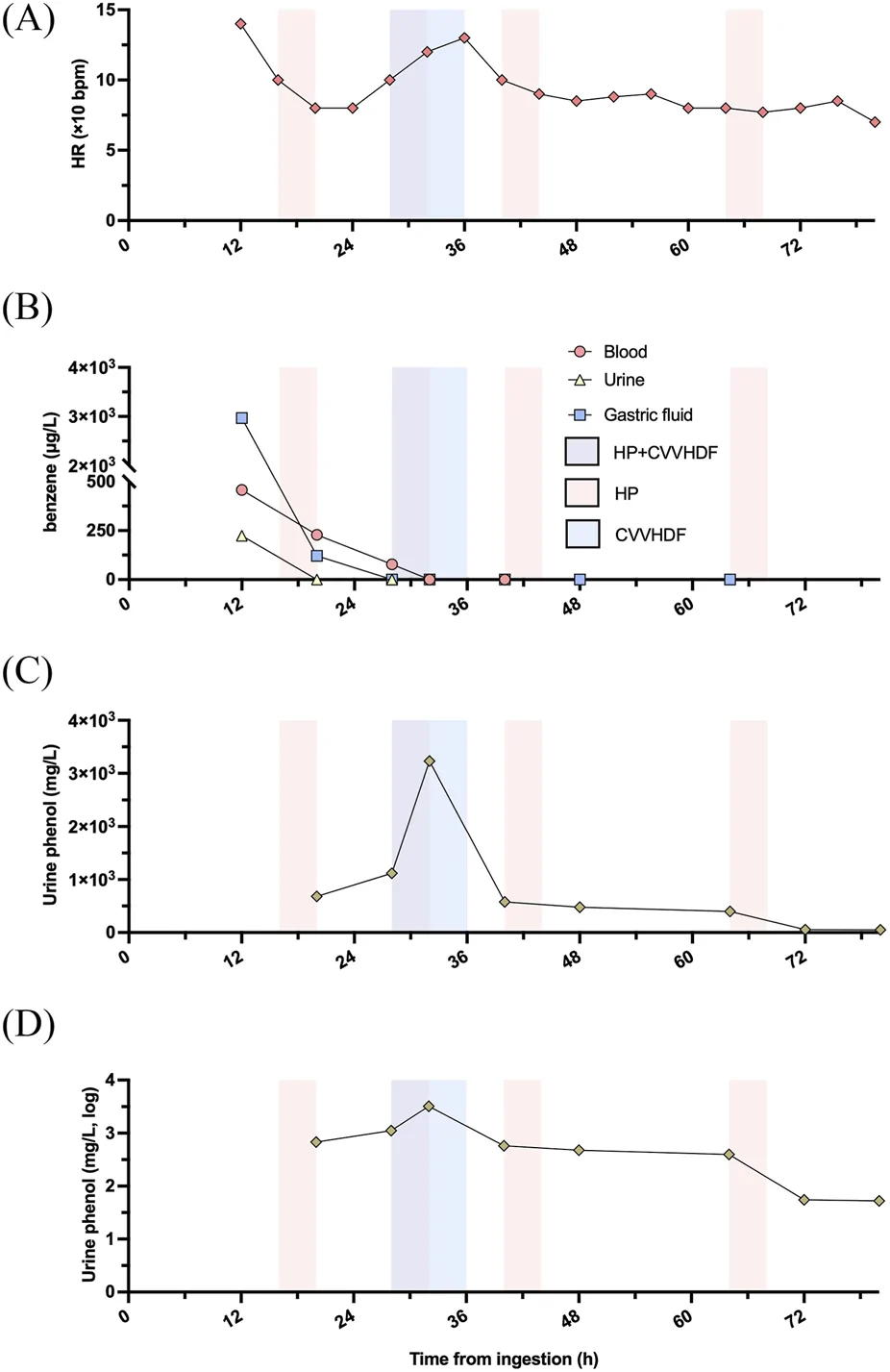
Clinical course, extracorporeal therapy, and toxicological trends after severe acute oral ingestion of a benzene-containing solvent. **(A)** Heart rate over time. **(B)** Benzene concentrations in gastric fluid, blood, and urine; filled markers denote measured concentrations and open markers denote values below the limit of detection (LOD), which are plotted at the matrix-specific LOD. Dashed horizontal reference lines indicate the LOD for blood (12 μg/L) and for gastric fluid and urine (3 μg/L). **(C)** Urinary phenol concentration over time (linear y-axis). **(D)** Log10-transformed urinary phenol concentration over time to display the wide dynamic range. Pink shaded vertical bars indicate hemoperfusion (HP) sessions; light-blue shaded vertical bars indicate continuous veno-venous hemodiafiltration (CVVHDF) periods. Light-purple shading indicates the treatment window during which HP and CVVHDF were performed simultaneously. Time on the x-axis is hours from ingestion. All numerical values are taken from Table 1. Plotting values below the LOD at the matrix-specific LOD is a visualization choice and may visually overestimate very low concentrations.

The timeline of events, extracorporeal interventions, and toxicological monitoring is summarized in Table 1 and Figure 1. Laboratory and imaging findings during hospitalization are summarized in Table 2.

**TABLE 1:** Timeline of exposure, symptoms, interventions, and toxicological monitoring from 0 to 108 h after ingestion.

Time from ingestion (h)	Event	HR (bpm)	Symptoms	Gastric fluid benzene (µg/L)	Urine benzene (µg/L)	Blood benzene (µg/L)	Urinary phenol (mg/L)
0	Ingestion of benzene-containing solvent (∼15.6 g benzene)	​	Transient loss of consciousness	​	​	​	​
12	Admission; pre-lavage toxicology sampling; subsequent skin decontamination, gastric lavage, catharsis; IV glucuronolactone, glutathione, and vitamin C	140	Chest tightness, abdominal pain, nausea, retching, fecal incontinence	2,966^a^	224^a^	457^a^	​
16	HP × 4 h	100	Slightly improved	​	​	​	​
20	Post-HP sample	80	Slightly improved	121	<3	228	681.2
24	Monitoring	80	Slightly improved	​	​	​	​
28	HP × 4 h + CVVHDF × 4 h	100	Nausea, retching, sore throat, dark urine	<3	<3	78	1119.4
32	CVVHDF × 4 h	120	Chest tightness, nausea, retching, sore throat, dark urine	<3	​	<12	3231.5
36	Furosemide 20 mg	130	Chest tightness, nausea, retching, sore throat, dark urine	​	​	​	​
40	HP × 4 h	100	Nausea, retching, sore throat, dark urine	​	​	​	577.8
44	Furosemide 20 mg	90	Sore throat, retching; urine clearing	​	​	​	478.5
48	​	85	Sore throat, abdominal pain	​	​	​	​
52	​	88	Sore throat, abdominal pain	​	​	​	​
56	​	90	Sore throat, abdominal pain	​	​	​	​
60	​	80	Sore throat, abdominal pain	​	​	​	​
64	HP × 4 h	80	Sore throat, abdominal pain	​	​	​	397.2
68	​	77	Improved	​	​	​	55.3
72	​	80	Improved	​	​	​	​
76	​	78	Improved	​	​	​	52.6
108	​	75	Improved	​	​	​	39.6

Abbreviations: CVVHDF, continuous veno-venous hemodiafiltration; HP, hemoperfusion; HR, heart rate; IV, intravenous. Values reported as “<3” or “<12” indicate concentrations below the relevant limit of detection. The limits of detection were 12 μg/L for blood and 3 μg/L for gastric fluid and urine. Blank cells indicate not available or not measured, and some missing samples resulted from logistical constraints.

^a^ The admission gastric fluid, blood, and urine samples were collected before gastric lavage.

**TABLE 2:** Laboratory and examination findings during hospitalization and follow-up.

Hospital day	WBC (×10^9^/L)	Hb (g/L)	LYM (×10^9^/L)	PT (s)	FIB (g/L)	ALT (U/L)	AST (U/L)	CK (U/L)	MYO (ng/mL)	Other examinations
0^a^	20.85	174	0.41	14.8	2.44	11	31	54	10.91	ECG: Sinus tachycardia, right axis deviation. Bedside cardiac and pulmonary ultrasound: no major abnormality. Emesis and stool: Occult blood positive
1	15.07	149	0.99	15.4	2.48	9	39	3786	1097.09	ECG: Sinus rhythm, right axis deviation
2	11.45	126	1.27	14.5	2.66	10	37	3122	41.51	​
3	​	​	​	​	​	​	​	​	​	Chest and abdominal CT: right lower lobe infection; cholestasis. Brain MRI: Scattered nonspecific punctate white-matter hyperintensities in bilateral frontal and parietal lobes
4	5.17	122	0.72	11.3	3.01	330	250	308	​	​
8	5.13	123	0.80	12.3	3.96	85	24	​	​	​
9	​	​	​	​	​	​	​	​	​	Fecal occult blood: Negative
15	​	​	​	​	​	​	​	​	​	Gastroscopy: Erosive gastritis. Brain MRI: Persistent scattered nonspecific punctate white-matter hyperintensities in bilateral frontal and parietal white matter
18	3.68	127	0.96	​	​	16	14	​	1.39	​
35	3.87	155	0.94	12.6	2.17	18	21	​	​	​
65	4.35	164	1.1	13.3	1.74	15	14	57	​	Brain MRI: Resolution of previous white-matter abnormalities
119	4.63	168	1.07	​	​	17	14	​	​	​

Abbreviations: ALT, alanine aminotransferase; AST, aspartate aminotransferase; CK, creatine kinase; CT, computed tomography; ECG, electrocardiogram; FIB, fibrinogen; Hb, hemoglobin; LYM, lymphocyte count; MRI, magnetic resonance imaging; MYO, myoglobin; PT, prothrombin time; WBC, white blood cell count. Blank cells indicate not available or not measured. Hospital day 0 corresponds to the day of admission, approximately 12 h after ingestion. ^a^ Hospital day 0 values were obtained on admission before gastric lavage, decontamination, extracorporeal treatment, or other specific therapeutic interventions.

## Methods

The patient was admitted to and clinically managed at Maternal and Child Health Hospital of Hubei Province. All toxicological measurements (benzene in whole blood, gastric fluid, and urine; assay-defined total urinary phenol) were performed at the clinical toxicology laboratory of Hubei Provincial Hospital of Integrated Chinese and Western Medicine, which served as the external reference toxicology laboratory; samples were collected at the bedside and transferred under standard conditions to the reference laboratory.

Benzene in blood, urine, and gastric fluid was measured using a PerkinElmer 680/SQ8T gas chromatography–mass spectrometry system (PerkinElmer, USA). For benzene determination, 1 mL of sample was transferred into a sealed headspace vial, followed by addition of 2 g sodium chloride and 1 mL distilled water. After sealing, the vial was incubated in a 60 °C water bath for 1 h, and 1 mL of headspace gas was injected for analysis. Chromatographic separation was performed on a PerkinElmer Elite-5MS capillary column (30 m × 0.25 mm × 0.25 μm). The oven temperature was held at 40 °C for 2 min and then increased to 120 °C at 10 °C/min. Helium was used as the carrier gas at 1 mL/min. Qualitative analysis was performed in full-scan mode (m/z 40–300), and quantitative analysis was performed in SIM mode using ions m/z 78, 63, and 51. The linear range was 0.012–2.0 mg/L, the correlation coefficient was 0.9995, spike recovery was 95.3%–104.2%, the limit of detection was 0.012 mg/L, and the lower limit of quantification was 0.036 mg/L.

Urinary phenol was measured using a Shimadzu GC-2030 gas chromatograph (Shimadzu, Japan). Briefly, 5 mL of urine was mixed with 1 mL of hydrochloric acid and heated in a 90 °C water bath for 1 h. After cooling, 3 mL of diethyl ether was added, the sample was shaken for 1 min, and the organic supernatant was analyzed by gas chromatography. The lower detection limit was 1.5 mg/L (based on 5 mL urine), the calibration range was 1.5–50 mg/L, the reported precision was 3.3%–5.4%, and spike recovery was 82.8%–87.0%. Samples above the upper calibration limit were diluted and reanalyzed, and the final concentrations were calculated after applying the corresponding dilution factor. This assay was interpreted as measuring assay-defined total urinary phenol after acid treatment and extraction rather than benzene-specific metabolites.

The initial gastric fluid, blood, and urine samples reported at admission were all collected before gastric lavage; therefore, these admission concentrations were not affected by lavage-related dilution. Subsequent samples were obtained during the post-decontamination course according to clinical feasibility. Sampling was scheduled before and after each HP or CVVHDF session whenever feasible; some time points were not sampled because of logistical constraints, and blank cells in Table 1 indicate values not available or not measured. Daily urine output during the first five hospital days was extracted retrospectively from the medical record, but urine creatinine was not measured and no residual urine samples remained for retrospective creatinine correction.

Routine biochemistry, hematology, coagulation, and cardiac enzyme testing were performed on the standard clinical analyzers of Maternal and Child Health Hospital of Hubei Province. Brain MRI was performed on a 1.5-T system and included axial T1-weighted, sagittal and axial T2-weighted, and T2 fluid-attenuated inversion recovery (FLAIR) sequences; image interpretation was provided by the on-duty neuroradiologist.

## Results

### Clinical course and organ injury

On admission (approximately 12 h after ingestion) the patient had sinus tachycardia (heart rate 140 beats/min), leukocytosis, mild coagulopathy, and gastrointestinal symptoms, but was hemodynamically stable and neurologically non-focal. Within the first 2 hospital days he developed rhabdomyolysis (creatine kinase peak 3786 U/L; myoglobin peak 1097.09 ng/mL on hospital day 1), with subsequent rapid decline of muscle injury markers after extracorporeal therapy and fluid management. Hepatocellular injury emerged on hospital day 4 (ALT 330 U/L, AST 250 U/L) and resolved over the following 1–2 weeks. Imaging documented right lower lobe pneumonia and cholestasis on hospital day 3, and gastroscopy performed on hospital day 15 confirmed erosive gastritis. Brain MRI on hospital day 3 already showed scattered small white-matter lesions in the bilateral frontal and parietal lobes; similar nonspecific lesions were again seen on repeat brain MRI on hospital day 15, and these findings normalized on follow-up imaging (Table 2).

### Serial benzene concentrations in biological matrices

Before gastric lavage and before the first extracorporeal session, benzene was detected simultaneously in gastric fluid (2,966 μg/L), blood (457 μg/L), and urine (224 μg/L), confirming the exposure and supporting ongoing intragastric and systemic toxic burden 12 h after ingestion (Table 1). Because the initial gastric fluid, blood, and urine samples were all collected before gastric lavage, the admission benzene concentrations cannot be attributed to lavage-related dilution and provide cleaner bedside evidence of persistent exposure at presentation.

After the first HP session (20 h after ingestion), gastric fluid benzene decreased to 121 μg/L and blood benzene to 228 μg/L; urinary benzene was below the limit of detection at this time point. Gastric fluid benzene became undetectable by 28 h. Blood benzene decreased further to 78 μg/L at 28 h and became undetectable by 32 h.

### Serial urinary phenol concentrations

In contrast to the falling blood benzene, urinary phenol rose after presentation. It increased from 681.2 mg/L at 20 h to 1119.4 mg/L at 28 h and peaked at 3231.5 mg/L at 32 h (Table 1; Figure 1). A stepwise decline followed, with urinary phenol concentrations of 577.8 mg/L at 40 h, 478.5 mg/L at 44 h, 397.2 mg/L at 64 h, 55.3 mg/L at 68 h, 52.6 mg/L at 76 h, and 39.6 mg/L at 108 h. These values represent uncorrected spot concentrations; because urine creatinine was not measured and no residual samples remained for retrospective correction, they should be interpreted as qualitative trend markers rather than creatinine-corrected quantitative excretion estimates.

Recorded urine output was 2,410 mL on hospital day 1, 3780 mL on hospital day 2, 3020 mL on hospital day 3, 4,360 mL on hospital day 4, and 3105 mL on hospital day 5; accordingly, the observed urinary phenol concentrations should be interpreted with caution because urine dilution may have influenced the measured values.

### Temporal relationship between toxicokinetic trends and clinical recovery

HP and CVVHDF sessions were delivered over the first three hospital days, during which blood benzene became undetectable and urinary phenol reached its peak and then began to decline. Additional HP sessions beyond the time when blood benzene became undetectable were continued pragmatically because of the magnitude of the reported ingestion, delayed presentation, persistent gastrointestinal symptoms, dark urine, evolving organ injury, and the contemporaneous rise and later decline of urinary phenol, rather than because of any proven efficacy after blood benzene had become undetectable. De-escalation of extracorporeal support coincided with a sustained downward trend in urinary phenol and with resolution of dark urine, overall improvement in heart rate after transient fluctuation during the early course, and improvement of gastrointestinal symptoms (Table 1).

## Discussion

This case describes a severe acute oral ingestion of a benzene-containing solvent in which serial measurement of both the parent compound and a major urinary metabolite was used to support clinical interpretation during hemoperfusion and CVVHDF. Temporal association in this single case does not imply causation, and the favorable outcome may have reflected the combined effects of decontamination, supportive care, extracorporeal therapy, natural recovery, or their interaction. The report is therefore intended to illustrate a clinical and toxicokinetic pattern rather than establish a treatment effect.

The case is unusual in two respects. First, the exposure route was oral and the dose substantial, whereas most benzene literature concerns inhalational occupational exposure or chronic hematotoxicity (Agency for Toxic Substances and Disease Registry, 2024; Mohammed et al., 2020). Second, serial monitoring demonstrated a temporal separation between disappearance of measurable blood benzene and delayed peaking of urinary phenol. In a patient presenting approximately 12 h after ingestion, this pattern is biologically plausible because benzene is rapidly absorbed, redistributed into lipid-rich tissues, and extensively biotransformed to hydroxylated metabolites, so the blood parent-compound concentration may not fully reflect evolving internal burden after redistribution and metabolism (Agency for Toxic Substances and Disease Registry, 2024; Zhang et al., 2024). However, the urinary phenol values in this case were uncorrected spot concentrations without urine creatinine correction or 24-h urinary collection and should therefore be interpreted as pragmatic trend markers rather than quantitative excretion estimates.

The peak urinary phenol concentration observed in this patient (3231.5 mg/L at 32 h) was substantially higher than reference values reported for occupational benzene exposure and for previously published clinical cases of acute benzene poisoning. Background concentrations in unexposed individuals are typically below 10 mg/L, and the ACGIH biological exposure index for end-of-shift urinary phenol following an 8-h benzene exposure of 10 ppm is approximately 50 mg/g creatinine (∼45–50 mg/L) (Inoue et al., 1986; Rahimpoor et al., 2023). In a previously reported case of acute benzene poisoning presenting with status epilepticus and extensive cerebral white-matter signal abnormalities, the urinary phenol concentration was 62 mg/L (Wang and Tao, 2021). The substantially higher peak observed in our patient is consistent with the magnitude of the ingested dose (∼15.6 g of benzene), which markedly exceeds typical occupational exposure scenarios from which existing reference ranges were derived. As a result, established occupational biological exposure indices were not directly applicable to this clinical setting, and the urinary phenol trajectory was used primarily as an internal trend marker for the individual patient rather than as a comparator against population-based thresholds.

In the present case, recurrent chest tightness and a second increase in heart rate were observed during the later course, around the time urinary phenol reached its peak. Although these findings were nonspecific, they raise the possibility that ongoing metabolite burden may have contributed to transient cardiovascular stress. The mechanisms of benzene-related cardiotoxicity are not fully established. Available evidence suggests that acute severe exposure may produce solvent-like central nervous system depression with possible arrhythmic and circulatory consequences. In addition, reactive benzene metabolites such as benzene oxide, hydroquinone, and trans,trans-muconaldehyde have been implicated in oxidative stress, endothelial activation, apoptosis, increased vascular permeability, and inflammatory cardiovascular injury, which may contribute to cardiac dysfunction and electrical instability (Zelko et al., 2025).

The clinical picture was consistent with severe systemic toxicity, including gastrointestinal injury, rhabdomyolysis, transient liver injury, right lower lobe pneumonia, and transient nonspecific white-matter abnormalities on MRI. These findings fit the known acute toxic profile of high-dose benzene exposure, although the contribution of benzene itself, the co-formulant ethyl acetate, aspiration, and secondary physiologic stress cannot be separated in a single case (Agency for Toxic Substances and Disease Registry, 2024). Because the ingested product was a benzene standard solution prepared in ethyl acetate rather than neat benzene, this case should be interpreted as a mixed-solvent ingestion with laboratory-confirmed benzene exposure. Ethyl acetate may have contributed to gastrointestinal irritation, transient central nervous system effects, and possibly to the nonspecific MRI findings. A recently published case of acute oral benzene poisoning complicated by multiple organ dysfunction also reported clinical recovery after intensive supportive management, supporting the feasibility of survival with aggressive, multimodal care in this high-mortality scenario, although the specific contribution of extracorporeal treatment could not be isolated in that report either (Wang, C., et al., 2025).

There are currently no benzene-specific recommendations from the EXTRIP workgroup. Therefore, extracorporeal treatment decisions in this case were based on general toxicology principles, the physicochemical properties of benzene and its metabolites, and the patient’s evolving clinical course. The role of extracorporeal treatment in benzene poisoning remains uncertain. Benzene is lipophilic and distributes rapidly into tissues, which may limit extracorporeal removal of the parent compound, and no benzene-specific EXTRIP recommendation is available (Agency for Toxic Substances and Disease Registry, 2024; Ghannoum et al., 2018). In this patient, HP and CVVHDF were not initiated on the basis of a single laboratory threshold; they were integrated into a broader bedside assessment that included the reported ingestion amount, delayed presentation, persistent measurable benzene, evolving organ injury, and later the downward trend in urinary phenol accompanied by clinical improvement. This framing is consistent with contemporary toxicology practice, in which hemoperfusion and CVVHDF are generally driven by the overall clinical picture rather than by a single concentration (Ghannoum et al., 2018). A similar strategy of dynamic toxicant monitoring to guide intensive care and extracorporeal management has recently been reported for other acute solvent and pesticide poisonings, suggesting that paired parent-compound and metabolite trajectories can add interpretive value at the bedside (Liu et al., 2025; Wang, K., et al., 2025).

From a clinical toxicology perspective, the combination of blood benzene and urinary phenol measurements was more informative than either marker alone. Blood benzene helped determine whether measurable parent compound was still present in the circulation, whereas urinary phenol signaled ongoing biotransformation and, subsequently, decline of metabolite burden. This paired approach may be particularly useful in late presenters, in whom parent-compound concentrations may already be falling but systemic complications are still emerging. Urinary phenol is not specific for low-level benzene exposure because it also arises from other sources, including diet and endogenous metabolism; in addition, uncorrected spot urinary concentrations are influenced by urine dilution and diuresis. Accordingly, in this laboratory-confirmed ingestion, urinary phenol was interpreted primarily as a pragmatic trend marker rather than a benzene-specific quantitative biomarker (Rahimpoor et al., 2023; Zhang et al., 2024).

The transient white-matter abnormalities on brain MRI also merit cautious interpretation. In our patient, the initial brain MRI on hospital day 3 already showed small punctate white-matter lesions in the bilateral frontal and parietal lobes, described radiologically as nonspecific demyelination-like changes; similar nonspecific lesions persisted on repeat MRI on hospital day 15 and subsequently resolved on follow-up imaging. Bilateral cerebral white-matter signal abnormalities have been previously described after acute benzene exposure, including a case in which extensive bilateral white-matter signal changes accompanied benzene-related status epilepticus and largely resolved after recovery (Wang and Tao, 2021). Nevertheless, these findings remain nonspecific. No cerebrospinal fluid analysis, oligoclonal bands, or additional demyelination workup was performed. Although no witnessed seizure was reported and the patient arrived awake with a GCS score of 14 while maintaining airway patency, transient loss of consciousness occurred before hospital arrival; therefore, brief hypoxia or secondary physiologic stress could also have contributed to the MRI findings. In addition, ethyl acetate exposure or unrelated incidental white-matter abnormalities cannot be excluded (Zheng et al., 2022).

This report has several important limitations, and its findings should be interpreted cautiously. First, it describes a single patient managed at one center; no inference can be drawn about the efficacy, safety, or optimal indications of hemoperfusion or CVVHDF in acute benzene-containing solvent ingestion, and the case only illustrates a clinical and toxicokinetic pattern rather than establishing a treatment effect. Second, quantitative exposure characterization depended on the product label and the history reported by the patient and family, without an independent product assay, so both the ingested benzene dose and the exact composition of the ingested solution carry uncertainty. Third, serial sampling was incomplete for some matrices and time points, and no plasma or effluent phenol concentrations were obtained; formal estimation of extracorporeal metabolite clearance, mass balance, or tissue redistribution was therefore not possible. Fourth, no urine creatinine measurements or 24-h urinary collections were available, and no residual urine samples remained for retrospective correction; urinary phenol could therefore not be normalized for urine dilution and was interpreted only as an uncorrected trend marker. Fifth, more specific benzene metabolites, such as S-phenylmercapturic acid and trans,trans-muconic acid, were not available in the local laboratory, and the proportion of urinary phenol directly attributable to benzene biotransformation in this patient cannot be determined with certainty (Rahimpoor et al., 2023; Zhang et al., 2024). Sixth, multiple co-interventions were applied in parallel (decontamination, hepatoprotective therapy, fluid management, antimicrobial therapy, hemoperfusion, and CVVHDF), and their individual contributions to the favorable outcome cannot be separated from the natural time course of recovery. Seventh, although additional analytical details have now been provided, full retrospective access to all validation parameters, including stability data and internal standard details, was not available. Finally, causal attribution of specific complications—in particular the transient white-matter abnormalities—to benzene itself, as opposed to the co-formulant ethyl acetate, transient hypoxia during the early post-ingestion period, or secondary physiologic stress, cannot be resolved in a single case. Longer hematologic surveillance is also advisable because benzene is leukemogenic, although in our patient the 1-year follow-up laboratory results remained normal. Our observations should therefore be regarded as hypothesis-generating and should not be used to inform treatment decisions in the absence of further prospective evaluation.

## Conclusion

Severe oral ingestion of a benzene-containing solvent is a rare, life-threatening form of acute solvent poisoning for which neither a specific antidote nor a benzene-specific extracorporeal treatment recommendation is available. In the case reported here, paired serial monitoring of blood benzene and urinary phenol during sequential hemoperfusion and CVVHDF provided a coherent toxicokinetic picture: the parent compound fell rapidly after admission, the metabolite peaked later and then declined, and de-escalation of extracorporeal support coincided with a sustained downward trend in the metabolite together with clinical improvement.

Although a single case cannot establish treatment efficacy, it illustrates a pragmatic monitoring framework that may help clinicians interpret late toxicokinetic trends and contextualize decisions regarding hemoperfusion and CVVHDF in severe acute solvent ingestions, in line with emerging experience from dynamic toxicant monitoring in other acute poisonings. Prospective series and standardized toxicological sampling protocols, including more specific benzene metabolites, are needed to better define the role of extracorporeal treatment and of paired parent-compound and metabolite biomarkers in this rare but high-risk clinical scenario.

## Patient perspective

At follow-up, the patient reported that the most distressing aspects of the illness were persistent nausea, throat discomfort, and uncertainty about the outcome during the first hospital days. He and his family expressed relief at the gradual recovery and at the absence of persistent neurologic symptoms. After psychiatric evaluation and psychological support, he returned to a normal mental state, and they agreed to publication of this case in the hope that it may help clinicians manage similar severe poisonings.

## Data Availability

The original contributions presented in the study are included in the article; further inquiries can be directed to the corresponding author.
